# A Transformer-Based Framework for Counterfactual Estimation of Antihypertensive Treatment Effect on COVID-19 Infection Risk - A Proof-of-Concept Study

**DOI:** 10.1093/ajh/hpaf055

**Published:** 2025-04-18

**Authors:** Tran Q B Tran, Stefanie Lip, Honghan Wu, Shyam Visweswaran, Jill P Pell, Sandosh Padmanabhan

**Affiliations:** School of Cardiovascular and Metabolic Health, University of Glasgow, Glasgow, UK; School of Cardiovascular and Metabolic Health, University of Glasgow, Glasgow, UK; Queen Elizabeth University Hospital, Glasgow, UK; School of Health and Wellbeing, University of Glasgow, Glasgow, UK; University of Pittsburgh School of Medicine, Pittsburgh, PA, USA; School of Health and Wellbeing, University of Glasgow, Glasgow, UK; School of Cardiovascular and Metabolic Health, University of Glasgow, Glasgow, UK; Queen Elizabeth University Hospital, Glasgow, UK

**Keywords:** antihypertensive agents, angiotensin-converting enzyme inhibitors, calcium channel blockers, beta blockers, thiazides, COVID-19, counterfactual inference, neural network, deep learning

## Abstract

**Background:**

Transformer-based neural networks excel in modelling high-dimensional, time-series data with complex dependencies. This proof-of-concept study applies a transformer-X-learner framework to estimate treatment effects using real-world data, using antihypertensive drug exposure and COVID-19 risk as an exemplar.

**Methods:**

We conducted a case-control study of 303,220 NHS Greater Glasgow and Clyde patients aged ≥ 40 years during the first two COVID-19 pandemic waves. Using a transformer-X-learner framework that incorporated temporal patterns in medication usage and comorbidities, we controlled for confounding effects and estimated individual and average treatment effects ACEIs, beta-blockers (BBs), calcium channel blockers (CCBs), thiazides (THZs), and statins on 180-day SARS-CoV-2 infection risk.

**Results:**

The transformer-X-learner framework outperformed traditional approaches, achieving an F1 score of 0.82 and area under the precision-recall curve (AUPRC) of 0.78. ACEIs showed a negligible overall impact on COVID-19 risk (ATE: 0.97%±5.5), while BBs (-8.3%±7.3%) and CCBs (-9.7%±8.1%) were protective. Statins (3.5%±6.1%) and THZs (4.3%±10.8%) showed slight increases in risk. Treatment effects were consistent across age, gender, and socioeconomic categories.

**Conclusions:**

ACEIs do not substantially increase the risk of COVID-19 infection while the protective effects of BBs and CCBs warrant further investigation. This study highlights the potential of transformer-based causal inference models as a powerful tool for evaluating treatment safety and efficacy in complex healthcare scenarios.

## Graphical Abstract

**Graphical Abstract:**
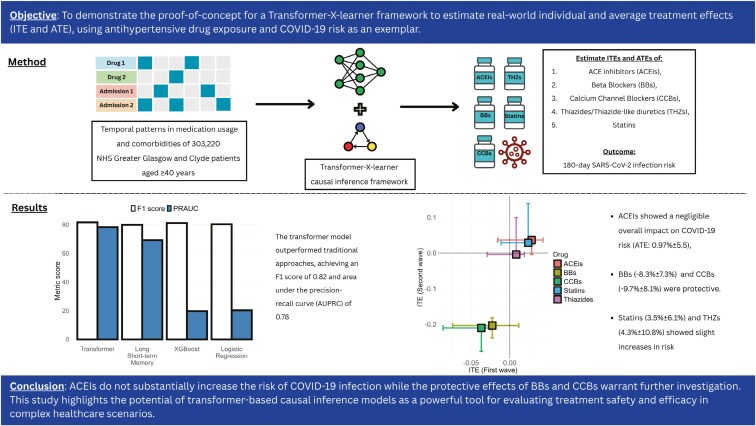

## Introduction

The COVID-19 pandemic prompted a rapid investigation into how common medications impact infection risk and disease severity. Concurrently, advances in artificial intelligence, especially deep learning, provided new approaches for modeling high-dimensional, temporal healthcare data. Transformer-based models, in particular, have shown superior ability to capture long-range dependencies in sequential data like patient medication histories and hospital admission records.^1,2^

Estimating individual (ITE) and average treatment effects (ATE) from observational data is a key challenge in pharmacological research. Traditional regression models often struggle with time-varying confounders and complex treatment patterns. In contrast, transformer architectures combined with counterfactual methods like the X-learner^3^ offer a robust framework for causal effect estimation in dynamic, temporally structured patient data.

This study demonstrates a proof-of-concept transformer-X-learner model for causal inference in drug safety surveillance. Using a large, diverse cohort from NHS Greater Glasgow and Clyde population, we evaluate the model’s ability to estimate treatment effects on COVID-19 infection risk. We focus on antihypertensive drugs, including angiotensin-converting enzyme inhibitors (ACEIs), beta-blockers (BBs), calcium channel blockers (CCBs), thiazides/thiazide-like diuretics (THZs), and statins, as clinically relevant examples due to their widespread use and previously debated links to COVID-19 outcomes.

ACEIs, commonly prescribed as first-line therapy for hypertension, heart failure, diabetes, and renal disease, confer cardiovascular and renal protection primarily by inhibiting the renin-angiotensin-aldosterone system (RAAS) and reducing levels of angiotensin II, a potent vasoconstrictor.^4–6^ Angiotensin-converting enzyme 2 (ACE2), a critical regulator within RAAS, counterbalances angiotensin II by converting it into the vasodilatory, anti-inflammatory peptide angiotensin 1-7.^7,8^

Intriguingly, ACE2 also serves as the cellular entry point for SARS-CoV-2, the virus responsible for the COVID-19 pandemic.^9^ The widespread expression of ACE2 on endothelial cells renders the vascular system susceptible to COVID-19-related injury,^10^ raising concerns regarding the cardiovascular safety of ACE inhibitors during the pandemic, as these medications may upregulate ACE2.^11^ Existing literature reports conflicting findings: early observational studies indicated higher mortality among hypertensive COVID-19 patients,^12,13^ while subsequent research suggested protective effects potentially mediated through ACE2 and angiotensin 1-7 upregulation, reducing inflammation, fibrosis, and thrombosis.^14,15^

Our primary objective is to validate the utility of a transformer-X-learner framework for causal inference in real-world settings. The secondary aim is to contribute to the ongoing discourse on the safety and potential protective effects of specific antihypertensive classes during infectious disease outbreaks. This methodological approach has broader implications for individualized treatment effect estimation across diverse therapeutic domains.

## Methods

This study is reported as per the Strengthening the Reporting of Observational Studies in Epidemiology (STROBE) guideline.^16^

### Study Population

The study population included patients who were 40 years of age and older as of October 1, 2019, selected due to its higher prevalence of hypertension and greater use of antihypertensive medications. Additionally, older adults have an increased risk of severe COVID-19 outcomes, highlighting the importance of understanding medication effects in this vulnerable population.

Patients with missing demographic data, specifically sex, age, or Scottish Index of Multiple Deprivation (SIMD), were excluded.

### NHS Greater Glasgow and Clyde Safe Haven

The West of Scotland Safe Haven is a trusted research environment that links and provides remote access to routinely collected healthcare datasets. Approvals to use reference data sets were obtained from the Safe Haven Local Privacy and Advisory Committee (LPAC).

### Description of Derived Variables

This study used linked data from four databases: The Prescribing Information System (PIS), the Scottish Morbidity Record 01 (SMR01), death certificates, and patient demographics. The PIS records all community-dispensed medications, coded using the British National Formulary (BNF). The SMR01 records hospital admission dates and diagnoses, coded using the International Classification of Diseases 10 (ICD-10). Death certificates provide dates and cause of death, also ICD-10 coded. Demographics data include patient age, sex, and area-based socioeconomic deprivation measured by the SIMD deciles (lower SIMD values indicate higher deprivation).

### Exposure

Exposure to each study medication was defined as one or more PIS dispensing records during two periods: October 1, 2019–April 1, 2020 (first wave) and April 1, 2020-October 1, 2020 (second wave). Medications, identified by BNF codes, included: ACEIs (0205051), BBs (0204000), CCBs (0206020), THZs (0202010), and statins (0212000B0, 0212000C0, 0212000X0, 0212000Y0).

### Follow-up and Outcomes

Separate analyses were performed for the first (from April 1, 2020) and second pandemic waves (from October 1, 2020), with the second wave aligning with the UK COVID-19 vaccination rollout from December 8, 2020. Due to the differences in vaccination status and evolving public health responses, analyzing the data for the first and second waves separately allows for a clearer understanding of the study medications’ impact on the COVID-19 infection risk.

To address the severe class imbalance favoring COVID-19-negative patients, which may cause bias in the models’ estimations, we performed undersampling on the COVID-19-negative group.^17,18^ We used a stratified sampling based on age, sex, and SIMD. The ratio was determined by dividing the number of COVID-19 cases observed during the first wave by the number of cases observed during the second wave. Patients excluded during this process formed an independent cohort for second-wave analysis, enabling separate evaluation of the two pandemic waves.

**Figure 1:**
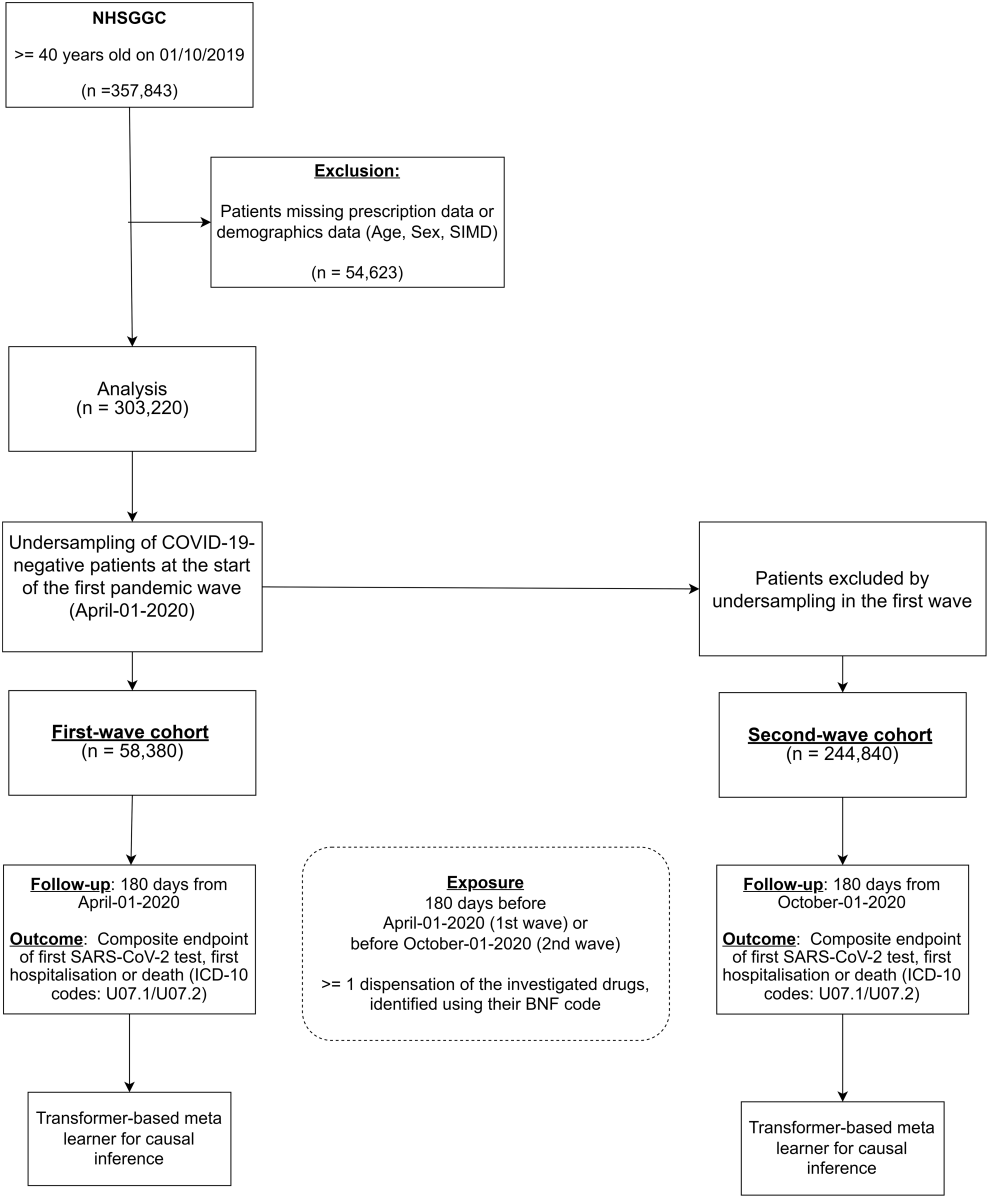
Flow diagram of the number of patients included in the analysis for the two pandemic waves. ACEIs: Angiotensin-converting-enzyme inhibitors, BBs: Beta blockers, CCBs: Calcium channel blockers,THZs: Thiazides and thiazide-like diuretics.

The outcome was incident COVID-19, a composite endpoint defined as the earliest of: first positive SARS-CoV-2 test, first COVID-19 hospitalization, or COVID-19 death (ICD-10 codes U07.1/U07.2). This was assessed within a 180-day period from the start of each wave. Follow-up continued until the first eligible event or the end of the follow-up period (October 1, 2020, for the first wave; April 1, 2021, for the second wave), whichever occurred first. (Figure 1, Supplementary Figure 1)

### Neural Network and Causal Inference Model

A transformer-based neural network predicted incident COVID-19 within a 180-day window using both time-invariant (age, sex, SIMD deciles) and time-dependent features. Time-dependent features comprised longitudinal prescription and comorbidity patterns within the 180-day period preceding each COVID-19 wave. Hospital admissions were represented by ICD-10 codes and medication dispenses by BNF codes.

The transformer architecture enhances prediction of 180-day COVID-19 infection by modeling interdependencies within and between a patient’s past prescriptions and hospital admissions. This approach enables the model to construct a context-aware representation that captures both short-term long-term trends in medications and admissions, thus improving predictive performance.

Static variables (age, sex, SIMD, and diabetes status) were concatenated to the output of the transformer model to form the final neural network model (Supplementary Figure 2).

The black-box nature of neural networks has hindered prior studies from robustly quantifying the causal effects of input covariates on outcome occurrence. To address this gap, we integrated the transformer model into the X-learner framework, a robust causal inference method, to estimate ITEs and ATEs for the aforementioned medications on 180-day COVID-19 infection risk. The X-learner trained separate models on treated and control groups, predicted counterfactual outcomes under opposite treatments, and then estimated ITEs, allowing for personalized effect estimation (Supplementary Figure 3).

Leveraging transformer as the base model for the X-learner algorithm facilitated adjustment for static covariates, such as age, sex, SIMD, and diabetes status, alongside longitudinal data on hospital admissions and medication dispenses within the 180-day period preceding each population, and thus accounting for the potential influence of background medications and comorbidities. Details of the transformer model and X-learner framework are presented in the extended method, and Supplementary Table 1&2.

### Individual treatment effect and average treatment effect

The ITE is the difference between the outcome an individual would experience with a treatment versus the outcome they would experience without it. ATE is the average difference in outcomes between treated and control groups and provides an estimate of the overall causal impact of the treatment in the population.^19^

### Ethics

Delegated research ethics approval was granted for linkage to National Health Service (NHS) patient data by the Local Privacy and Advisory Committee at NHS Greater Glasgow and Clyde. Cohorts and de-identified linked data were prepared by the West of Scotland Safe Haven Research Database at NHS Greater Glasgow and Clyde.

### Statistical analyses

We evaluated the model’s 180-day COVID-19 infection predictions using 5-fold cross-validation, assessing accuracy, F1-score, and Area Under the Precision-Recall Curve (AUPRC) for each pandemic wave. AUPRC was used to evaluate the trade-off between precision and recall across classification thresholds, making it suitable for imbalanced classification. We compared the performance of our transformer-based model to other classification methods including logistic regression, XGBoost, and Long Short-term Memory (LSTM).

Cohort demographics were summarized using medians and interquartile ranges (IQRs) for continuous variables (e.g., age) and frequencies and percentages for categorical variables (e.g., gender, SIMD deciles, diabetes status).

Wilcoxon signed-rank tests compared paired ITEs between ACEIs and each other drug category (BBs, CCBs, THZs, statins). The magnitude of differences in ITEs between ACEIs and the other drugs was assessed using the effect size, estimated by the correlation coefficient (*r*). Effect sizes were classified as small (0.1 ≤ *r* < 0.3), medium (0.3 ≤ *r* < 0.5), or large (r ≥ 0.5), providing an interpretation of the clinical significance independent of sample size.

Conditional density plots were used to visualize ITE distribution variations across covariates (age, gender, SIMD decile).

Statistical analyses were conducted using R (v4.3.3) and Python (v3.8).

## Results

The analysis included 303,220 participants: 58,380 (19.3%) from the first wave and 244,840 (80.7%) from the second. Median age was 60.5 years (first wave: 61.5; second wave: 60.2), 56.2% (170,263) were female, and 22.8% (68,102) were in the highest SIMD decile (Table 1). Incident COVID-19 within 180 days of wave onset was 9.1% (5,344 patients) in the first wave and 9.6% (23,521 patients) in the second. Table 1 summarises the ten most frequent medications and hospital admission causes during the 180-day period preceding each wave. Supplementary Tables 3&4 summarise patient characteristics by study drugs across both COVID-19 waves.

**Table 1:** Overall patient demographics for the first and second COVID-19 pandemic wave. The table includes the top 10 dispensed medications and comorbidities from prior hospital admissions for the overall study population. All other comorbidities and medications, although not shown in the table, were also incorporated as inputs for the neural network model.*Incident COVID-19 is a composite endpoint comprising the first positive SARS-CoV-2 test result, the first hospitalisation, or death specifically attributed to COVID-19 (ICD-10 codes U07.1/U07.2) within a 180-day period from the start of the first wave or the second wave

		First Wave	Second Wave	Total
Total N (%)		58,380 (19.3)	244,840 (80.7)	303,220
Age	Median (IQR)	61.5 (52.2 to 73.3)	60.2 (51.4 to 72.0)	60.5 (51.5 to 72.3)
Sex (n, % patient population in the respective wave)	Female	32,982 (56.5)	137,281 (56.1)	170,263 (56.2)
	Male	25,398 (43.5)	107,559 (43.9)	132,957 (43.8)
SIMD deciles (n, % patient population in the respective wave)	1	13,078 (22.7)	55,024 (22.8)	68,102 (22.8)
	2	8,560 (14.9)	34,993 (14.5)	43,553 (14.6)
	3	5,207 (9.1)	22,236 (9.2)	27,443 (9.2)
	4	4,559 (7.9)	19,578 (8.1)	24,137 (8.1)
	5	4,004 (7.0)	16,409 (6.8)	20,413 (6.8)
	6	3,755 (6.5)	15,623 (6.5)	19,378 (6.5)
	7	3,553 (6.2)	14,358 (5.9)	17,911 (6.0)
	8	4,303 (7.5)	17,665 (7.3)	21,968 (7.4)
	9	5,672 (9.9)	24,760 (10.3)	30,432 (10.2)
	10	4,816 (8.4)	20,679 (8.6)	25,495 (8.5)
Diabetes status (n, % patient population in the respective wave)	0	53,660 (91.9)	224,920 (91.9)	278,580 (91.9)
	1	4,720 (8.1)	19,920 (8.1)	24,640 (8.1)
Dispensed medications (top 10 shown) (n, % patient population in the respective wave)	Proton pump inhibitors	24,946 (42.7)	112,635 (46.0)	137,581 (45.4)
	Non-opioid analgesics	20,905 (35.8)	98,623 (40.3)	119,528 (39.4)
	Lipid-regulating drugs	19,270 (33.0)	79,672 (32.5)	98,942 (32.6)
	Beta blockers	12,092 (20.7)	51,873 (21.2)	63,965 (21.1)
	Selective beta^2^ agonists	10,529 (18.0)	47,839 (19.5)	58,368 (19.2)
	Angiotensin-converting enzyme inhibitors	11,369 (19.5)	47,833 (19.5)	59,202 (19.5)
	Antiplatelets	11,358 (19.4)	46,394 (18.9)	57,752 (19.0)
	Calcium channel blockers	10,680 (18.3)	44,993 (18.4)	55,673 (18.4)
	Broad-spectrum penicillin	8,440 (14.5)	44,659 (18.2)	53,099 (17.5)
	Selective serotonin reuptake inhibitors	10,215 (17.5)	44,432 (18.1)	54,647 (18)
Comorbidities from prior hospital admissions (top 10 shown) (n, % of patient population in the respective wave)	No admission	44,653 (76.5)	212,745 (86.9)	257,398 (84.9)
	Cataract	828 (1.42)	654 (0.27)	1,482 (0.49)
	Unspecified acute lower respiratory infection	389 (0.67)	619 (0.25)	1,008 (0.33)
	Urinary tract infection	374 (0.64)	999 (0.41)	1,373 (0.45)
	Chronic obstructive pulmonary dieases	280 (0.48)	534 (0.22)	814 (0.27)
	Atheroslerotic heart disease	272 (0.47)	774 (0.32)	1,046 (0.34)
	Unspecified sepsis	229 (0.39)	617 (0.25)	846 (0.28)
	Malignant breast neoplasm	206 (0.35)	633 (0.26)	839 (0.28)
	Unspecified chest pain	187 (0.32)	717 (0.29)	904 (0.30)
	Syncope and collapse	140 (0.24)	429 (0.19)	569 (0.19)
Incident COVID-19*	0	53,036 (90.9)	221,319 (90.4)	274,355 (90.5)
	1	5,344 (9.1)	23,521 (9.6)	28,865 (9.5)

### Model Performance

The performance of the four models (Transformer, logistic regression, XGBoost, LSTM) across the two pandemic waves are presented in Table 2. The Transformer model demonstrated a strong overall performance, with the highest F1 score (first wave/second wave—0.816 ± 0.009/0.826 ± 0.012) and AUPRC (0.783 ± 0.001/0.779 ± 0.002) among the models.

**Table 2:** Performance metrics of Transformer model, logistic regression, XGBoost, and Long Short-term Memory (LSTM) for predicting incident COVID-19 within 180 days from the start of the first and second waves of the COVID-19 pandemic. All models were evaluated using accuracy, F1 score, and area under the precision-recall curve (AUPRC). All results were shown as percentage (± standard deviation). LSTM: Long short-term memory, AUPRC: Area under the precision-recall curve.

	Metrics	Transformer	Logistic Regression	XGBoost	Long Short-term Memory
First wave % (± SD)	Accuracy	77.2 (± 1.4)	79.1 (± 0.2)	78.3 (± 0.2)	75.4 (± 3.2)
	F1 score	81.6 (± 0.9)	80.3 (± 1.2)	81.1 (± 1.2)	79.9 (± 9.0)
	AUPRC	78.3 (± 0.1)	20.3 (± 3.5)	19.7 (± 3.2)	69.2 (± 4.0)
Second wave % (± SD)	Accuracy	78.5 (± 2.1)	80.8 (± 0.1)	79.9 (± 0.1)	76.3 (± 2.7)
	F1 score	82.6 (± 1.2)	81.2 (± 1.5)	82.1 (± 1.9)	80.4 (± 9.8)
	AUPRC	77.9 (± 0.2)	22.0 (± 4.1)	21.4 (± 1.7)	61.5 (± 1.8)

### ITE Analyses

**Figure 2:**
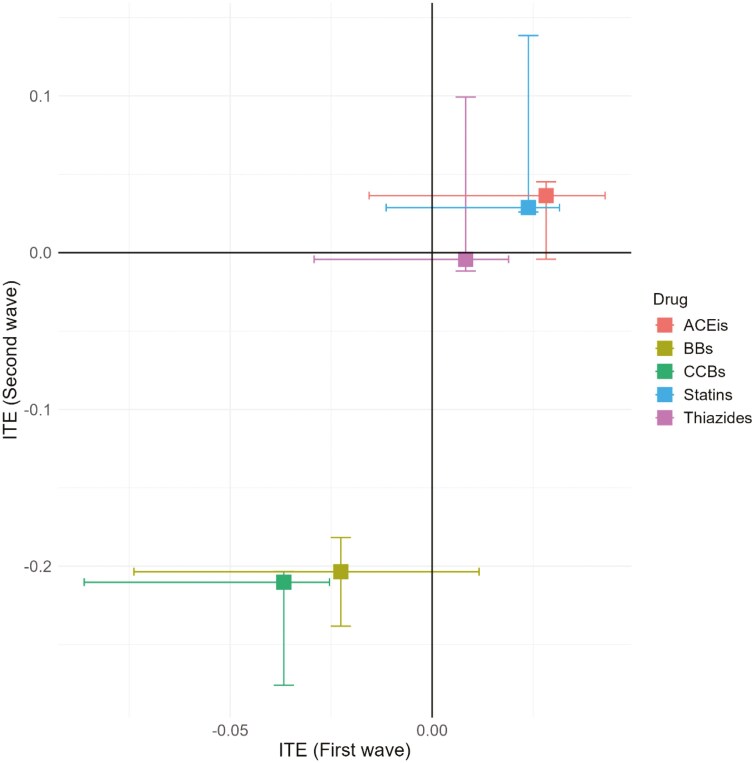
Individual Treatment Effects for different study drugs in the first (X-axis) and second (Y-axis) COVID-19 pandemic wave. ACEIs: Angiotensin-converting-enzyme inhibitors, BBs: Beta blockers, CCBs: Calcium channel blockers, ITE: individual treatment effect

During the first wave, ITE analyses revealed that patients taking ACEIs experienced a slight increase in COVID-19 risk, with a median risk difference of 2.8% (interquartile range [IQR]: -1.6% to 4.3%) (Figure 2, Supplementary Table 5). THZs showed negligible effect with a median risk difference of 0.8% (IQR: -2.9% to 1.9%). Statins were associated with slight increases in COVID-19 risk, with median risk differences of 2.4% (IQR: -1.1% to 3.2%). In contrast, BBs and CCBs showed protective effects against COVID-19. The median risk reductions were -2.3% (IQR: -7.4% to 1.2%) for BBs and -3.7% (IQR: -8.6% to -2.5%) for CCBs (Figure 2, Supplementary Table 5).

During the second wave, the ITEs of most drug classes mirrored those observed in the first wave (Figure 2, Supplementary Table 5). ACEIs showed a slight increase in infection risk, with a median increase of 3.6% (IQR: -0.4% to 4.5%). Statins were again associated with a small increase in risk, with median increases of 2.9% (IQR: 2.6% to 13.9%). THZs continued to show a negligible effect, with a median change of –0.4% (IQR: –1.2% to 9.9%). In contrast, BBs and CCBs remained associated with significant protective effects, with median risk reductions of -20.4% (IQR: -23.8% to -18.2%) and -21.0% (IQR: -27.6% to -20.3%), respectively.

### Weighted Average Treatment Effect Analyses

**Figure 3:**
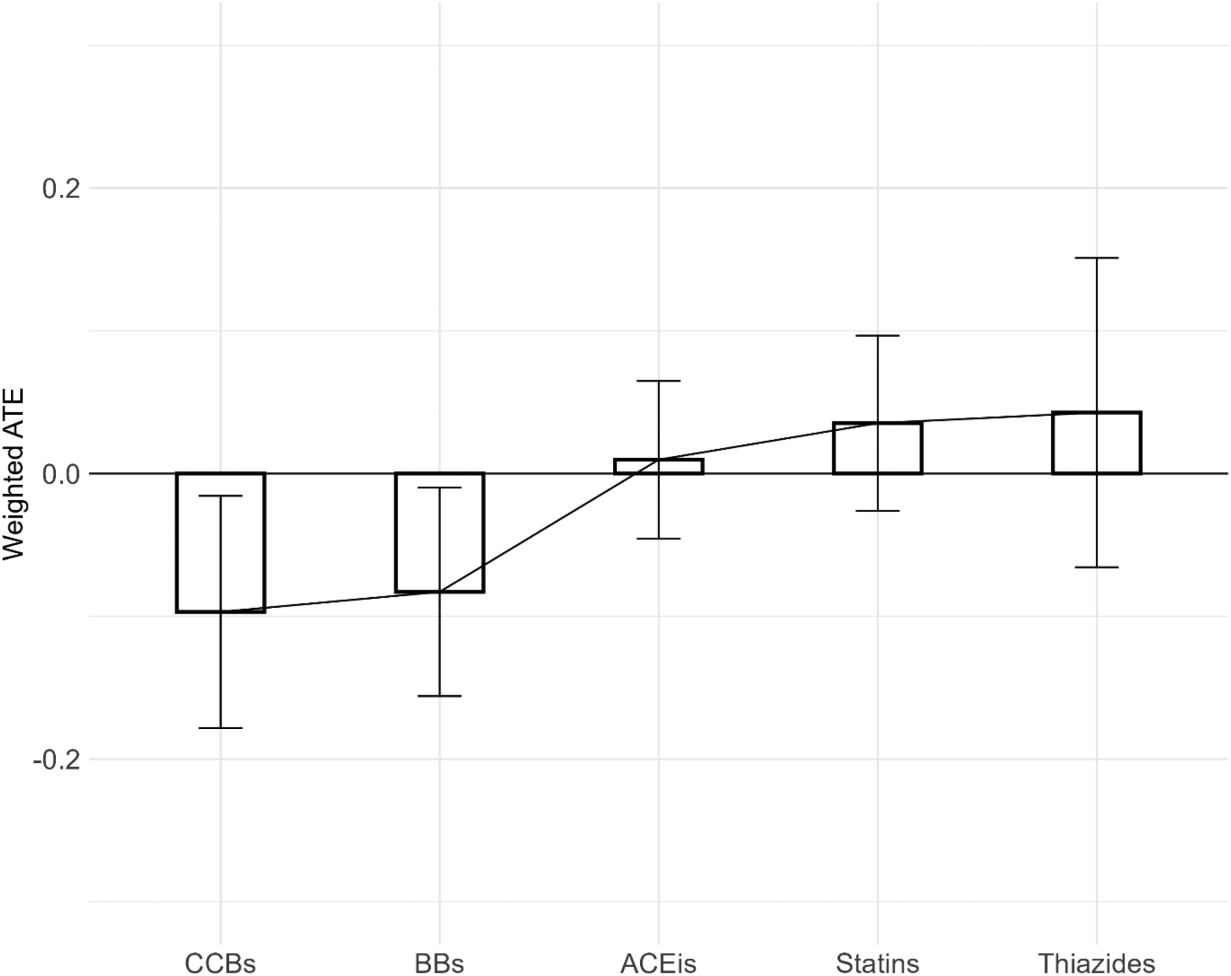
Overall weighted average treatment effect for study drugs. ACEIs: Angiotensin-converting-enzyme inhibitors, ATE: average treatment effect, BBs: Beta blockers, CCBs: Calcium channel blockers

Weighted average treatment effect (ATE) analyses reinforced these findings. Both statins (ATE: 3.5% ± 6.1%) and THZs (ATE: 4.3% ± 10.8%) were associated with increased risks, while ACEIs occupied a neutral position (ATE: 0.97% ± 5.5%). BBs (-8.3% ± 7.3%) and CCBs (-9.7% ± 8.1%) continued to exhibit protective effects (Figure 3, Supplementary table 6).

### Paired ITE Analyses—ACEI versus other drug classes

Paired analyses across both waves revealed significant differences between ACEIs and other drug classes (Table 3&Table 4). BBs and CCBs consistently exhibited large protective effects compared to ACEIs. For THZs, the effect size was moderate in the first wave but diminished to small in the second wave. Statins consistently demonstrated small effect sizes.

**Table 3:** Pair-wise analysis of ITEs between ACEIs and each of the other drug categories (BBs, CCBs, THZs, statins) for the first COVID-19 pandemic wave. Cohens d is a standardized effect size for measuring the difference between two group means. The effect size (r) is a measure of the magnitude of the relative ranks of paired differences. Effect sizes were classified as small (0.1 ≤ r < 0.3), medium (0.3 ≤ r < 0.5), or large (r ≥ 0.5). ACEIs: Angiotensin-converting-enzyme inhibitors, BBs: Beta blockers, CCBs: Calcium channel blockers, ITE: individual treatment effect, THZs: Thiazides and thiazide-like diuretics.

First Treatment	Second Treatment	First treatment’s ITE, median (IQR)	Second treatment’s ITE, median (IQR)	Cohens D (C.I.)	Wilcoxon signed-rank tests’ effect size	Effect size’s magnitude
ACEis	BBs	0.034 (0.044)	-0.018 (0.083)	-0.43 (-0.44, -0.42)	0.602	Large
ACEis	CCBs	0.034 (0.039)	-0.032 (0.039)	-0.48 (-0.49, -0.43)	0.611	Large
ACEis	THZs	0.033 (0.042)	0.012 (0.042)	-0.26 (-0.27, -0.25)	0.497	Moderate
ACEis	Statins	0.034 (0.039)	0.026 (0.032)	0.13 (0.12, 0.14)	0.202	Small

**Table 4:** Pair-wise analysis of ITEs between ACEIs and each of the other drug categories (BBs, CCBs, THZs, statins) for the second COVID-19 pandemic wave. Cohens d is a standardized effect size for measuring the difference between two group means. The effect size (r) is a measure of the magnitude of the relative ranks of paired differences. Effect sizes were classified as small (0.1 ≤ r < 0.3), medium (0.3 ≤ r < 0.5), or large (r ≥ 0.5). ACEIs: Angiotensin-converting-enzyme inhibitors, BBs: Beta blockers, CCBs: Calcium channel blockers, ITE: individual treatment effect, THZs: Thiazides and thiazide-like diuretics

First Treatment	Second Treatment	First treatment’s ITE, median (IQR)	Second treatment’s ITE, median (IQR)	Cohens D (C.I.)	Wilcoxon signed-rank tests’ effect size	Effect size’s magnitude
ACEis	BBs	0.039 (0.035)	-0.202 (0.052)	-1.62 (-1.75, -1.61)	0.794	Large
ACEis	CCBs	0.039 (0.033)	-0.208 (0.037)	-2.04 (-2.23, -2.03)	0.829	Large
ACEis	THZs	0.038 (0.035)	-0.006 (0.069)	0.22 (0.24, 0.24)	0.128	Small
ACEis	Statins	0.039 (0.029)	0.028 (0.030)	0.40 (0.44, 0.44)	0.096	Small

### Conditional Density Plots

Conditional density plots of ITEs across age, gender, and SIMD categories revealed consistent treatment effects across these demographic and socioeconomic groups. This uniformity suggests that the impact of the studied drug classes on COVID-19 infection risk does not vary significantly based on age, gender, or socioeconomic status (Supplementary figures 4-6).

## Discussion

This study, using data from over 300,000 patients, demonstrates the feasibility of integrating transformer-based models with causal inference to estimate treatment effects from real-world healthcare data. By modelling longitudinal medication use and hospitalisations within an X-learner framework, we introduce a scalable method for assessing both ITEs and ATEs across diverse patient cohorts.

The transformer model outperformed traditional models in AUPRC, which is vital for imbalanced datasets, likely by capturing detailed temporal interactions such as prescription sequences and comorbidity diagnoses. These findings support the suitability of transformer-based architectures for clinical prediction and causal modelling.

Although the clinical findings, neutral effects for ACEIs and potential protective effects for BBs and CCBs, align with prior literature, the study’s primary importance is on validating the transformer-X-learner framework. Our model reliably replicated known population-level associations and demonstrated consistent treatment effect distributions across demographic subgroups, underscoring its potential as a decision-support tool in pharmacovigilance and risk stratification.

Our study observed a marginally increased risk of COVID-19 infection in patients using ACEIs, a finding that aligns with both the biological mechanism of ACE2 upregulation, and previous observational researches advocating for continued ACEI use. A systematic review^20^ found that although ACEIs can influence ACE2 levels, the increase is limited and unlikely to significantly impact disease severity. Furthermore, large observational studies and meta-analyses^21–24^ generally show no major effect on severe outcomes or mortality in hypertensive patients using ACE inhibitors, supporting their continued use despite the slight infection risk. Our findings thus reinforce the safety of ACEIs during the COVID-19 pandemic and support current clinical guidelines for hypertensive patients.

BBs and CCBs were associated with protective effects against COVID-19 infection, with consistent findings across both pandemic waves. These findings may reflect their mechanisms of action, including modulation of immune responses^25^ and attenuation of ACE2 expression^26^ by BBs and inhibition of calcium-dependent viral entry pathways and post-entry replication events of SARS-CoV-2 in vitro by CCBs.^27,28^ Our findings are supported by previous observational studies that have shown beneficial effects of BB and CCBs.^28–31^ The robustness of these effects across demographic and socioeconomic groups highlights their potential for therapeutic repurposing.

The relationship between THZs, statins, and COVID-19 infection risk requires careful examination. While some studies suggest THZs may slightly increase COVID-19 risk through effects on sodium balance and RAAS system,^32–34^ others report a reduced risk of mortality in COVID-19 patients using THZs.^35^ Similarly, research on statins presents conflicting results. A study by Daniels et al.^36^ indicated statin use prior to hospital admission reduced the risk of severe COVID-19, while other studies suggested an elevated risk due to modulation of ACE2 expression and immune responses.^37,38^ Our findings of an association between THZs, statins, and increased COVID-19 risk underscore the need for further investigation. A deeper understanding of the underlying mechanisms, such as thiazide modulation of RAAS and statin impact on cholesterol metabolism, is essential for determining their clinical relevance in the context of the pandemic.

We acknowledge that other COVID-19 prediction models based on symptoms,^39^ biomarkers, or imaging data^40^ may outperform models based solely on administrative prescription data. However, our model addresses a unique niche: it enables real-time drug safety analysis in systems where structured clinical variables are limited, but medication and hospitalisation data are routinely available.

### Strengths and Limitations

By embedding the transformer within the X-learner framework, the model gains several advantages. The transformer’s positional embeddings capture temporal dynamics to adjust for the timing and sequence of medication use and health events, while the X-learner estimates ITEs, revealing variability often overlooked by traditional methods. Our study also showed consistent findings across different drugs in both waves, highlighting the model’s generalizability

Despite its strengths, several limitations should be acknowledged. The inherent complexity and black-box nature of transformers may hinder clinical adoption, as clinicians and policymakers often require interpretable outputs to trust and act on model outputs. Although advanced, the model cannot completely eliminate residual confounding, particularly from unmeasured variables. Another limitation is the lack of ethnicity and vaccination data, which may hinder clinical interpretability. Addressing these challenges will be critical for the broader adoption of such models.

Looking ahead, the transformer-X-learner framework can be extended to study treatment effects across multiple therapeutic areas beyond hypertension. Integration with explainability tools (e.g., SHAP, LIME) and real-time health system feeds may further enhance its clinical applicability. Second, expanding the model to more diverse populations, including broader ranges of ethnicity and age, would improve its generalizability and reliability. Finally, integrating additional dynamic covariates, such as real-time laboratory parameters, clinical symptoms, or vaccination status, would further strengthen the model’s predictive performance.

Given our study’s observational design and the challenges of Randomized Controlled Trials (RCTs) during a pandemic, future research on BBs and CCBs should emphasize pragmatic and mechanistic strategies. Pragmatic observational studies using high-quality real-world data (such as target trial emulation) can mimic RCT conditions while addressing time-varying confounding and treatment heterogeneity. Mendelian randomisation may elucidate causal links between genetic proxies for beta-adrenergic or calcium signalling and viral susceptibility. Furthermore, mechanistic studies, including in vitro and in vivo experiments, can explore cellular effects of beta-blockers and CCBs on viral entry, immune modulation, and endothelial function in the context of SARS-CoV-2. Finally, adaptive platform trials could incorporate these medications as potential arms, enabling real-time evidence generation without large-scale standalone RCTs.

## Conclusions

This study not only provides critical evidence on the differential impacts of antihypertensive drugs on COVID-19 infection risk but also highlights the transformative potential of machine learning in digital medicine. In conclusion, this study establishes a robust AI-driven methodology for causal inference in healthcare, validated in a timely and clinically relevant scenario. As digital health infrastructure matures, such approaches offer a powerful complement to traditional research, enabling continuous, data-driven refinement of treatment guidelines.

## Supplementary Data

Supplementary materials are available at *American Journal of Hypertension* (http://ajh.oxfordjournals.org).

hpaf055_suppl_Supplementary_Figures_1-6_Tables_1-6

## Data Availability

The data underlying this article were provided by the West of Scotland Safe Haven by permission. Data will be shared on request to the corresponding author with permission of the West of Scotland Safe Haven.
