# Supplementary material for: A Transformer-Based Framework for Counterfactual Estimation of Antihypertensive Treatment Effect on COVID-19 Infection Risk - A Proof-of-Concept Study
Source: Am J Hypertens. 2025 Apr 18;38(8):595–604. doi: 10.1093/ajh/hpaf055 (PMC12260164; doi:10.1093/ajh/hpaf055)
Supplement: hpaf055_suppl_Supplementary_Figures_1-6_Tables_1-6 [file hpaf055_suppl_supplementary_figures_1-6_tables_1-6.docx]

# Supplementary Figures

[Supplementary figure 1 Lookback and followup windows for first and second COVID-19 pandemic wave study cohorts 2](#_Toc195516053)

[Supplementary figure 2 An example of the data flow in the model for the first pandemic wave. The same data flow was performed for the second wave. 3](#_Toc195516054)

[Supplementary figure 3 Data flow in the X-learner pipeline.. 4](#_Toc195516055)

[Supplementary figure 4 Conditional density plot of weighted individual treatment effects by age. 5](#_Toc195516056)

[Supplementary figure 5 Conditional density plot of weighted individual treatment effects by sex. 6](#_Toc195516057)

[Supplementary figure 6 Conditional density plot of weighted individual treatment effects by SIMD deciles. 7](#_Toc195516058)

Supplementary figure 1 Lookback and followup windows for first and second COVID-19 pandemic wave study cohorts


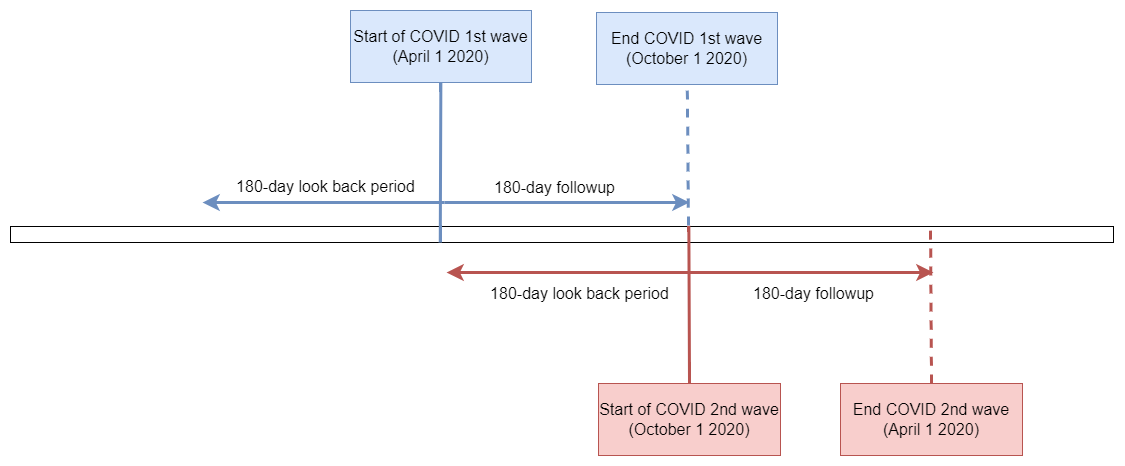


Supplementary figure 2 An example of the data flow in the model for the first pandemic wave. The same data flow was performed for the second wave. Hospital admissions (ICD-10 codes) and medication dispenses (BNF codes) 180-day prior to the start of the two COVID-19 pandemic waves were inputted as temporal sequences, with time-series of each variable flattened and tokenized into high-dimensional vectors. Temporal information was incorporated using positional embeddings that encoded the month and year of each event. Embedded tokens were processed through a transformer model's multi-head attention layer to capture key features and interactions across time points. Static variables (age, sex, SIMD, diabetes status) were input into a multilayer perceptron (MLP). Outputs from both models were concatenated to enable either classification or regression tasks. * Incident COVID-19 includes the first positive SARS-CoV-2 test, first hospitalisation, or COVID-19-related death (ICD-10 codes U07.1/U07.2) within 180 days from the start of the first wave or the second wave. SIMD: Scottish Index of Multiple Deprivation


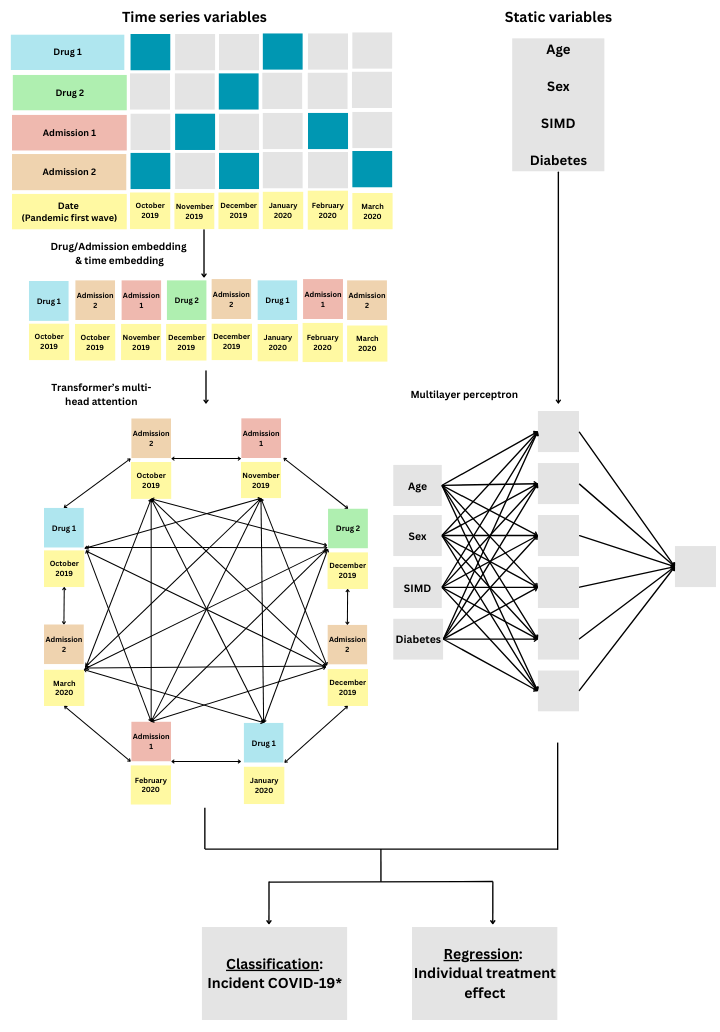


Supplementary figure 3 Data flow in the X-learner pipeline. The transformer model was used in the X-learner algorithm to enable adjustment for longitudinal data of background medications and comorbidities 180-day prior to the start of the two COVID-19 pandemic waves, as well as static variables (age, sex, SIMD, diabetes status). Training of the X-learner involved two stages. Stage 1 involved training transformer-based classifiers to predict incident COVID for the treated and control groups. These models were applied to the opposite groups to estimate counterfactual outcomes and calculate ITEs as the difference between actual and counterfactual outcomes. In Stage 2, transformer-based regressors were trained to predict ITEs for the treated and control groups and then applied to the entire population. Final ITEs were calculated as propensity-weighted averages of the Stage 2 predictions. * Incident COVID-19 includes the first positive SARS-CoV-2 test, first hospitalisation, or COVID-19-related death (ICD-10 codes U07.1/U07.2) within 180 days from the start of the first wave or the second wave.


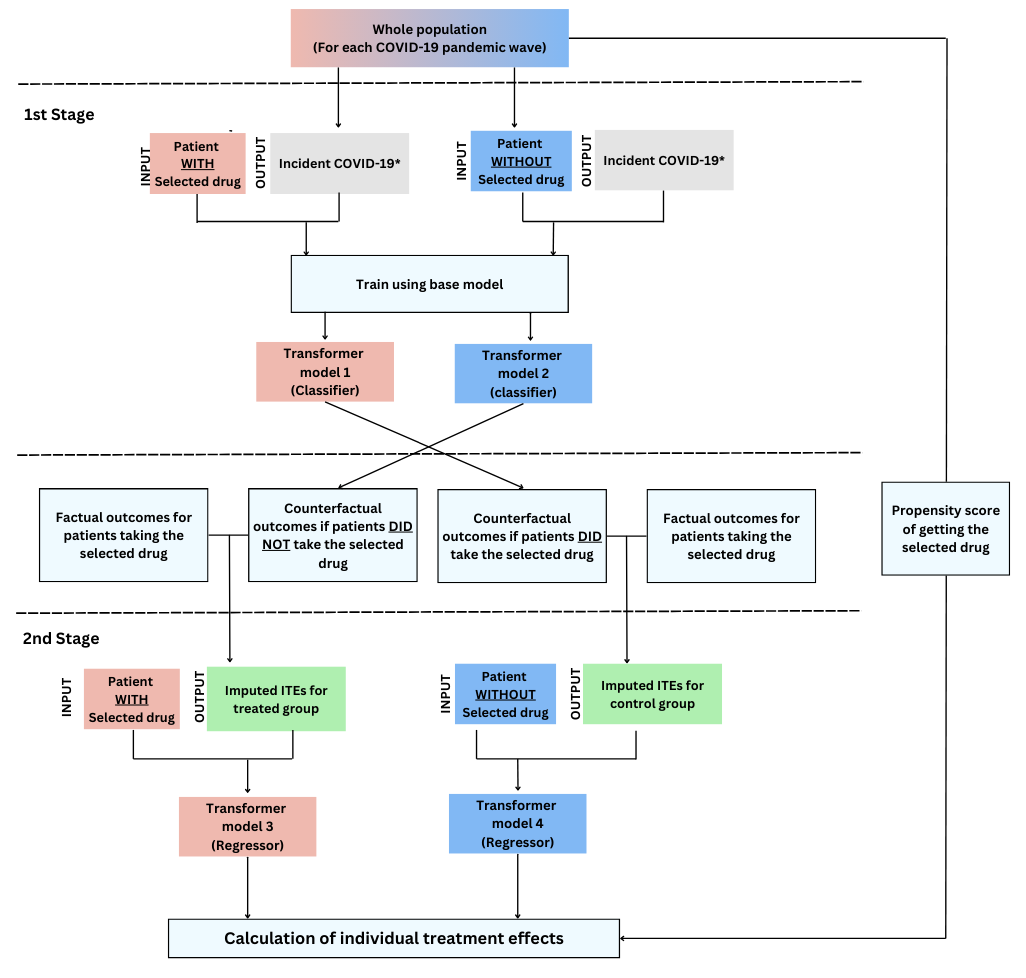


Supplementary figure 4 Conditional density plot of weighted individual treatment effects by age. ACEIs: Angiotensin-converting-enzyme inhibitors, BBs: Beta blockers, CCBs: Calcium channel blockers, ITE: Individual treatment effect, THZs: Thiazide-like diuretic


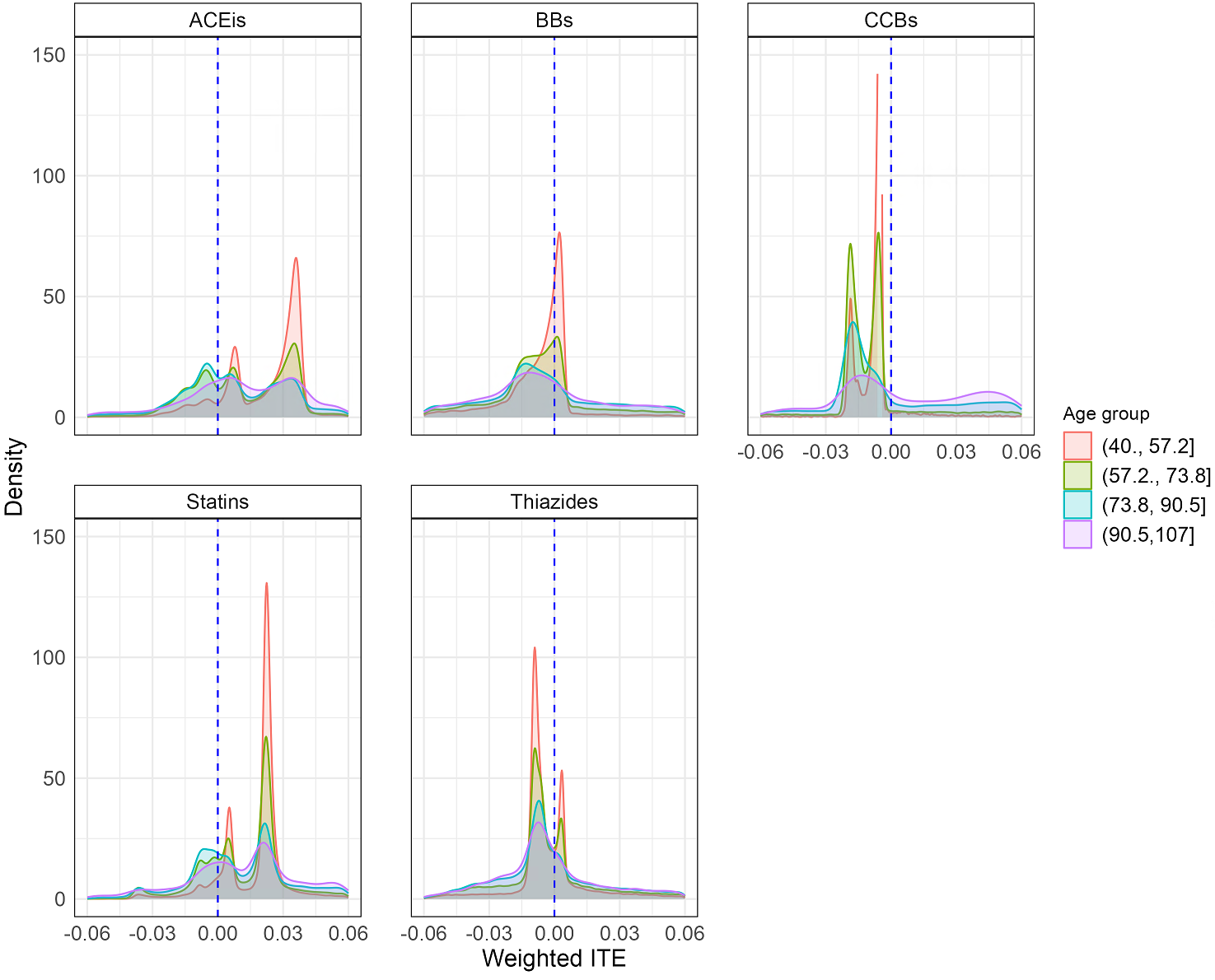


Supplementary figure 5 Conditional density plot of weighted individual treatment effects by sex. ACEIs: Angiotensin-converting-enzyme inhibitors, BBs: Beta blockers, CCBs: Calcium channel blockers, ITE: Individual treatment effect, THZs: Thiazide-like diuretic


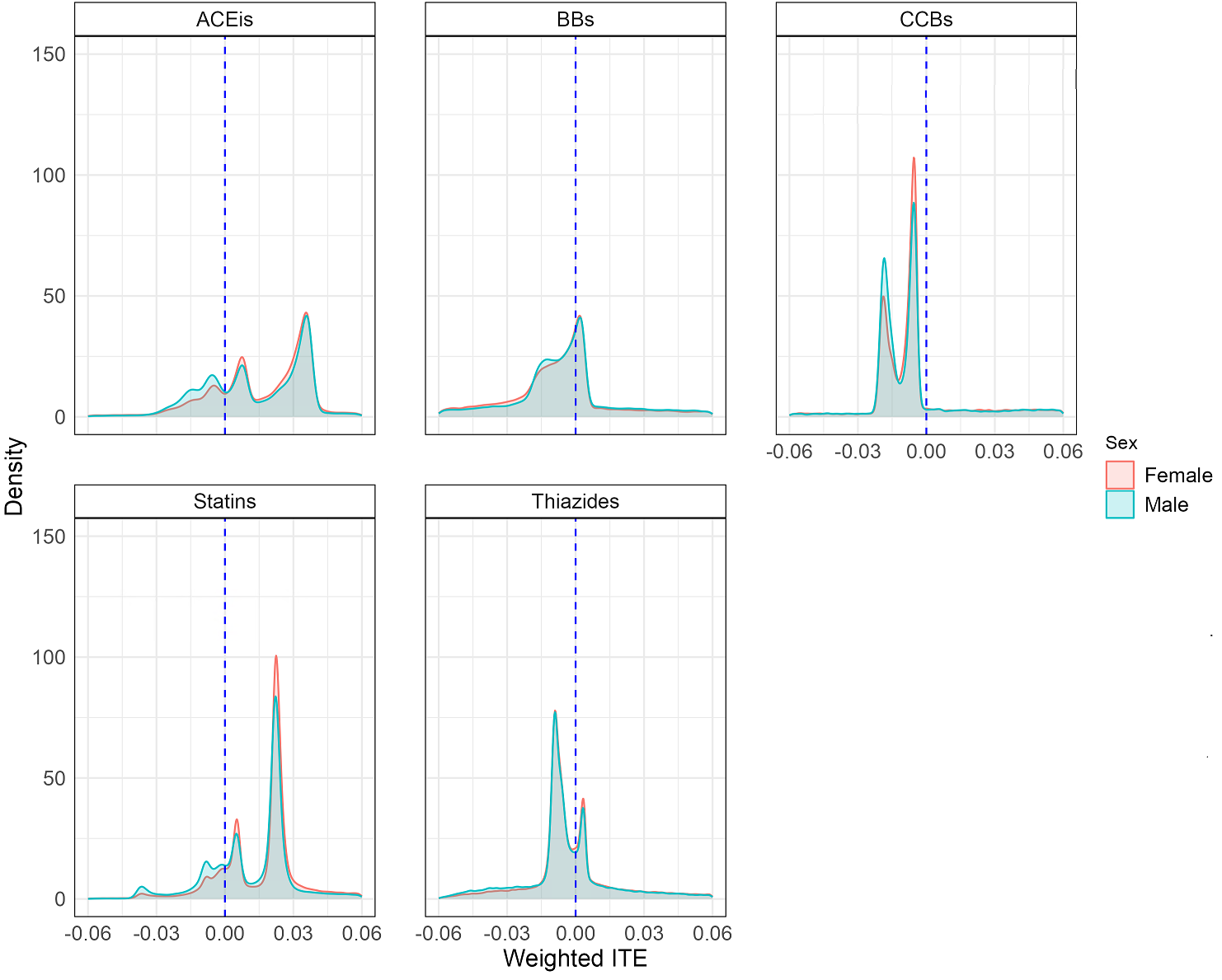


Supplementary figure 6 Conditional density plot of weighted individual treatment effects by SIMD deciles. ACEIs: Angiotensin-converting-enzyme inhibitors, BBs: Beta blockers, CCBs: Calcium channel blockers, ITE: Individual treatment effect, THZs: Thiazide-like diuretic


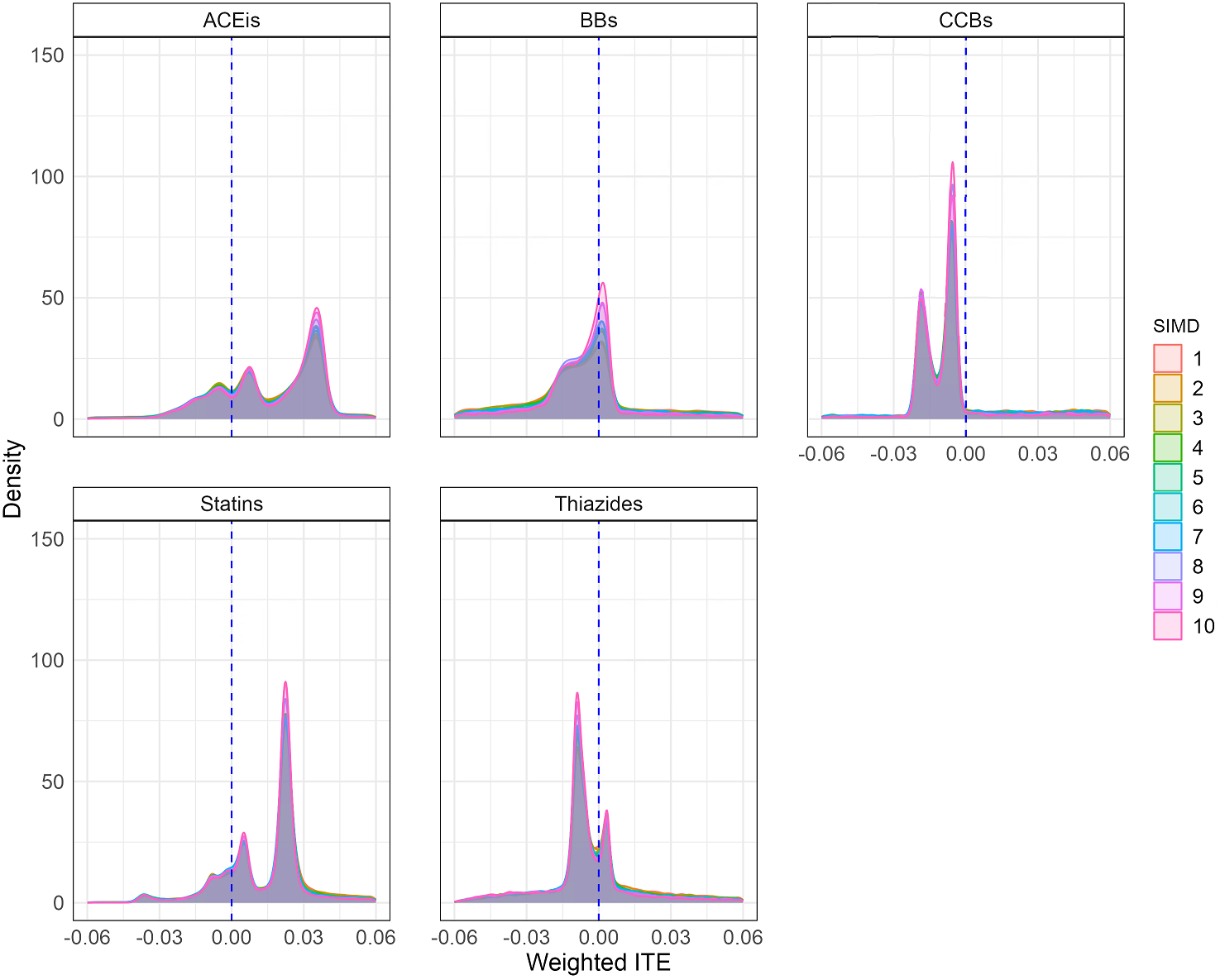


# Supplementary Tables

[Supplementary table 1 Core components of transformer-based neural network model](#_Toc195516059)

[Supplementary table 2 Training stages of X-former framework](#_Toc195516060)

[Supplementary table 3 Patient characteristics stratified by the study drugs for the first COVID-19 pandemic wave.](#_Toc195516061)

[Supplementary table 4 Patient characteristics stratified by the study drugs for the second COVID-19 pandemic wave.](#_Toc195516062)

[Supplementary table 5 Median ITE for study medications, stratifed by the two pandemic waves.](#_Toc195516063)

[Supplementary table 6 Weighted ATE for study medications, aggregated across both pandemic waves.](#_Toc195516064)

Supplementary table 1 Core components of transformer-based neural network model

| **Main Components of Model** | **Description** |
| --- | --- |
| Preprocessing encoding pipeline | To condense the raw high-dimensional time series data into a rich compact vector and encode the spatiotemporal data into a suitable format for the neural network |
| Transformer-based architecture with self-attention mechanism | To capture the spatiotemporal patterns, present in the electronic health record data |
| Feed-forward multilayer perceptron | To process the static and numerical data |
| Causal inference system (X-learner) | To estimate the causal effect of the selected drug to the risk of hospitalisation/death from COVID-19 |

Supplementary table 2 Training stages of X-former framework

| **Training Stage** | **Description** |
| --- | --- |
| First Stage | Two ML models (Model 1 and 2) were trained on time-series medication, admission and demographic data to predict the occurrence of COVID-19 related admissions/mortality in the preceding 6 months. Model 1 and 2 were trained on patients from the treated and control group respectively.  Subsequently, each model was cross-applied to the opposing group to generate predictions.  This purpose of this method is to obtain estimated counterfactuals by predicting what the treated group would have obtained, had they received the control and what the control group would have obtained when they received treatment.  Calculations of ITE will be computed based on counterfactual predictions.  Treated group: ITE represents the difference between the actual outcomes and counterfactual outcomes predicted by the control model  Control group: ITE corresponds to the difference between the counterfactual outcomes predicted by the treated model (from Stage 1) and actual outcome |
| Second Stage | Model 3 and 4 were built to predict the ITE of the control and treated group, respectively, using the same data inputs in the Stage-One Models (Model 1 and 2).  Models 3 and 4 were subsequently applied to input features from the entire population.  By imputing ITEs for both treated and untreated groups, a counterfactual scenario is created for each individual to allow us to compare outcomes under both treatment and no treatment. |
| Third Stage | Final ITEs were be estimated by obtaining the propensity-score-weighted average of Stage 2 models for each individual.  ATE for each medication is the average ITE across the whole population. |

Supplementary table 3 Patient characteristics stratified by the study drugs for the first COVID-19 pandemic wave. Medication counts represent the number of patients exposed to each drug, either as monotherapy or in combination with others. Percentages for each medication were calculated relative to the total study population of 58,380 patients in the first pandemic wave. All 58,380 patients were used in the transformer model to estimate the individual treatment effect of each drug. The table includes the top 10 dispensed medications and comorbidities from prior hospital admissions for the overall study population. All other comorbidities and medications, although not shown in the table, were also incorporated as inputs for the neural network model. *Incident COVID-19 is a composite endpoint comprising the first positive SARS-CoV-2 test result, the first hospitalisation, or death specifically attributed to COVID-19 (ICD-10 codes U07.1/U07.2) within a 180-day period from the start of the first wave. ACEIs: Angiotensin-converting-enzyme inhibitors, BBs: Beta blockers, CCBs: Calcium channel blockers, THZs: Thiazide-like diuretic.

|  |  | **ACEis** | **BBs** | **CCBs** | **THZs** | **Statins** |
| --- | --- | --- | --- | --- | --- | --- |
| Total N (%) |  | 11369 (19.5) | 12092 (20.7) | 10680 (18.3) | 4247 (7.3) | 19270 (33.0) |
| Age | Median (IQR) | 67.0 (58.0 to 76.5) | 68.9 (58.2 to 79.2) | 69.5 (60.7 to 78.0) | 70.1 (61.5 to 77.9) | 70.5 (61.6 to 78.8) |
| Sex | Female | 5143 (45.2) | 6351 (52.5) | 5422 (50.8) | 2592 (61.0) | 9160 (47.5) |
|  | Male | 6226 (54.8) | 5741 (47.5) | 5258 (49.2) | 1655 (39.0) | 10110 (52.5) |
| SIMD decile | 1 | 2726 (24.3) | 2856 (23.9) | 2396 (22.7) | 895 (21.3) | 4863 (25.6) |
|  | 2 | 1763 (15.7) | 1893 (15.9) | 1605 (15.2) | 653 (15.5) | 3005 (15.8) |
|  | 3 | 1095 (9.7) | 1166 (9.8) | 1005 (9.5) | 381 (9.1) | 1818 (9.6) |
|  | 4 | 884 (7.9) | 970 (8.1) | 886 (8.4) | 345 (8.2) | 1467 (7.7) |
|  | 5 | 787 (7.0) | 820 (6.9) | 742 (7.0) | 295 (7.0) | 1282 (6.7) |
|  | 6 | 735 (6.5) | 808 (6.8) | 668 (6.3) | 261 (6.2) | 1233 (6.5) |
|  | 7 | 675 (6.0) | 681 (5.7) | 640 (6.1) | 256 (6.1) | 1122 (5.9) |
|  | 8 | 772 (6.9) | 815 (6.8) | 758 (7.2) | 328 (7.8) | 1229 (6.5) |
|  | 9 | 1032 (9.2) | 1135 (9.5) | 1031 (9.8) | 450 (10.7) | 1698 (8.9) |
|  | 10 | 766 (6.8) | 787 (6.6) | 802 (7.6) | 345 (8.2) | 1297 (6.8) |
| Diabetes status | 0 | 9532 (83.8) | 10497 (86.8) | 9278 (86.9) | 3660 (86.2) | 15728 (81.6) |
|  | 1 | 1837 (16.2) | 1595 (13.2) | 1402 (13.1) | 587 (13.8) | 3542 (18.4) |
| Dispensed medications (top 10 in the overall patient population shown)  (n, % of population who are taking the index drug) | Proton pump inhibitors | 5608 (49.3) | 6616 (54.7) | 5387 (50.4) | 2034 (47.9) | 2034 (47.9) |
|  | Non-opioid analgesics | 4549 (40.0) | 5306 (43.9) | 4485 (42) | 1779 (41.9) | 1779 (41.9) |
|  | Lipid-regulating drugs | 7280 (64.0) | 7296 (60.3) | 6254 (58.6) | 2425 (57.1) | 2425 (57.1) |
|  | Beta blockers | 4313 (37.9) | 12092 (100) | 2947 (27.6) | 1227 (28.9) | 1227 (28.9) |
|  | Selective beta(2) agonists | 2039 (17.9) | 1809 (1 5) | 2119 (19.8) | 775 (18.2) | 775 (18.2) |
|  | ACE inhibitors | 11369 (100) | 4313 (35.7) | 4152 (38.9) | 1886 (44.4) | 1886 (44.4) |
|  | Antiplatelets | 4409 (38.8) | 5204 (43) | 3573 (33.5) | 1234 (29.1) | 1234 (29.1) |
|  | Calcium channel blockers | 4152 (36.5) | 2947 (24.4) | 10680 (100) | 1897 (44.7) | 1897 (44.7) |
|  | Broad-spectrum penicillin | 1617 (14.2) | 1838 (1 5.2) | 1630 (15.3) | 631 (14.9) | 631 (14.9) |
|  | Selective serotonin reuptake inhibitors | 1778 (15.6) | 2381 (19.7) | 1556 (14.6) | 591 (13.9) | 591 (13.9) |
| Comorbidities from prior hospital admissions (top 10 in the overall patient population shown)  (n, % of population who are taking the index drug) | No admission | 8276 (72.8) | 8287 (68.5) | 7682 (71.9) | 3198 (75.3) | 13405 (69.6) |
|  | Cataract | 230 (2.02) | 261 (2.16) | 236 (2.21) | 120 (2.83) | 458 (2.38) |
|  | Unspecified acute lower respiratory infection | 74 (0.65) | 113 (0.93) | 74 (0.69) | 18 (0.42) | 181 (0.94) |
|  | Urinary tract infection | 75 (0.66) | 121 (1.01) | 61 (0.57) | 21 (0.49) | 181 (0.94) |
|  | Chronic obstructive pulmonary dieases | 56 (0.49) | 74 (0.61) | 60 (0.56) | 9 (0.21) | 150 (0 78) |
|  | Atheroslerotic heart disease | 160 (1.41) | 223 (1.84) | 89 (0.83) | 21 (0.49) | 248 (1.29) |
|  | Unspecified sepsis | 59 (0.52) | 79 (0.65) | 38 (0.36) | 9 (0.21) | 108 (0.56) |
|  | Malignant breast neoplasm | 32 (0.28) | 27 (0.22) | 27 (0.25) | 12 (0.28) | 37 (0.19) |
|  | Unspecified chest pain | 73 (0.64) | 105 (0.87) | 68 (0.64) | 12 (0.28) | 126 (0.65) |
|  | Syncope and collapse | 41 (0.36) | 50 (0.41) | 30 (0.28) | 14 (0.33) | 82 (0.43) |
| Incident COVID-19* | 0 | 10498 (92.3) | 11020 (91.1 ) | 9869 (92.4) | 3984 (93.8) | 17636 (91.5) |
|  | 1 | 871 (7.66) | 1072 (8.87) | 811 (7.59) | 263 (6.19) | 1634 (8.48) |

Supplementary table 4 Patient characteristics stratified by the study drugs for the second COVID-19 pandemic wave. Medication counts represent the number of patients exposed to each drug, either as monotherapy or in combination with others. Percentages for each medication were calculated relative to the total study population of 244,840 patients in the second pandemic wave. All 244,840 patients were used in the transformer model to estimate the individual treatment effect of each drug. The table includes the top 10 dispensed medications and comorbidities from prior hospital admissions for the overall study population. All other comorbidities and medications, although not shown in the table, were also incorporated as inputs for the neural network model.*Incident COVID-19 is a composite endpoint comprising the first positive SARS-CoV-2 test result, the first hospitalisation, or death specifically attributed to COVID-19 (ICD-10 codes U07.1/U07.2) within a 180-day period from the start of the second wave. ACEIs: Angiotensin-converting-enzyme inhibitors, BBs: Beta blockers, CCBs: Calcium channel blockers, THZs: Thiazide-like diuretic.

|  |  | **ACEIs** | **BBs** | **CCBs** | **THZs** | **Statins** |
| --- | --- | --- | --- | --- | --- | --- |
| Total N (%) |  | 47833 (19.5) | 51873 (21.2) | 44993 (18.4) | 17867 (7.3) | 79672 (32.5) |
| Age | Median (IQR) | 66.0 (57.1 to 75.8) | 67.3 (56.7 to 77.5) | 68.7 (59.9 to 77.5) | 70.0 (61.1 to 77.7) | 69.7 (61.0 to 77.9) |
| Sex | Female | 21526 (45.0) | 27386 (52.8) | 22692 (50.4) | 10994 (61.5) | 37818 (47.5) |
|  | Male | 26307 (55.0) | 24487 (47.2) | 22301 (49.6) | 6873 (38.5) | 41854 (52.5) |
| SIMD decile | 1 | 11379 (24.1) | 12456 (24.3) | 10208 (23.0) | 3815 (21.6) | 20110 (25.5) |
|  | 2 | 7230 (15.3) | 8006 (15.6) | 6555 (14.7) | 2532 (14.3) | 12413 (15.8) |
|  | 3 | 4661 (9.9) | 4969 (9.7) | 4331 (9.7) | 1775 (10.0) | 7678 (9.8) |
|  | 4 | 3903 (8.3) | 4260 (8.3) | 3642 (8.2) | 1424 (8.0) | 6278 (8.0) |
|  | 5 | 3265 (6.9) | 3589 (7.0) | 3122 (7.0) | 1258 (7.1) | 5360 (6.8) |
|  | 6 | 3001 (6.3) | 3256 (6.4) | 2860 (6.4) | 1098 (6.2) | 4815 (6.1) |
|  | 7 | 2731 (5.8) | 2941 (5.7) | 2650 (6.0) | 1108 (6.3) | 4448 (5.6) |
|  | 8 | 3206 (6.8) | 3373 (6.6) | 3156 (7.1) | 1284 (7.3) | 5108 (6.5) |
|  | 9 | 4398 (9.3) | 4748 (9.3) | 4374 (9.8) | 1902 (10.8) | 6996 (8.9) |
|  | 10 | 3509 (7.4) | 3618 (7.1) | 3572 (8.0) | 1497 (8.5) | 5528 (7.0) |
| Diabetes status | 0 | 39725 (83.0) | 45047 (86.8) | 38820 (86.3) | 15431 (86.4) | 64548 (81.0) |
|  | 1 | 8108 (17.0) | 6826 (13.2) | 6173 (13/7) | 2436 (13.6) | 15124 (19.0) |
| Dispensed medications (top 10 in the overall patient population shown)  (n, % of population who are taking the index drug) | Proton pump inhibitors | 25137 (52.6) | 30069 (58) | 24489 (54.4) | 9417 (52.7) | 46836 (58.8) |
|  | Non-opioid analgesics | 21942 (45.9) | 25405 (49) | 21458 (47.7) | 8677 (48.6) | 40277 (50.6) |
|  | Lipid-regulating drugs | 31195 (65.2) | 31060 (59.9) | 26510 (58.9) | 10424 (58.3) | 79672 (100) |
|  | Beta blockers | 18837 (39.4) | 51873 (100) | 13148 (29.2) | 5609 (31.4) | 31060 (39) |
|  | Selective beta(2) agonists | 9410 (19.7) | 8711 (16.8) | 9954 (22.1) | 3622 (20.3) | 18204 (22.8) |
|  | ACE inhibitors | 47833 (100) | 18837 (36.3) | 18347 (40.8) | 7922 (44.3) | 31195 (39.2) |
|  | Antiplatelets | 18768 (39.2) | 21949 (42.3) | 15091 (33.5) | 5191 (29.1) | 38982 (48.9) |
|  | Calcium channel blockers | 18347 (38.4) | 13148 (25.3) | 44993 (100) | 8665 (48.5) | 26510 (33.3) |
|  | Broad-spectrum penicillin | 8821 (18.4) | 10337 (19.9) | 8730 (19.4) | 3350 (18.7) | 16680 (20.9) |
|  | Selective serotonin reuptake inhibitors | 7502 (15.7) | 10987 (21.2) | 6989 (15.5) | 2815 (15.8) | 14010 (17.6) |
| Comorbidities from prior hospital admissions (top 10 in the overall patient population shown)  (n, % of population who are taking the index drug) | No admission | 39803 (83.2) | 41737 (80.5) | 37360 (83.0) | 15143 (84.7) | 65098 (81.7) |
|  | Cataract | 182 (0.38) | 197 (0.38) | 217 (0.48) | 81 (0.45) | 332 (0.42) |
|  | Unspecified acute lower respiratory infection | 145 (0.30) | 233 (0.45) | 158 (0.35) | 36 (0.20) | 330 (0.41) |
|  | Urinary tract infection | 250 (0.52) | 323 (0.62) | 250 (0.56) | 93 (0.52) | 516 (0.65) |
|  | Chronic obstructive pulmonary dieases | 109 (0.23) | 117 (0.23) | 121 (0.27) | 28 (0.16) | 247 (0.31) |
|  | Atheroslerotic heart disease | 490 (1.02) | 647 (1.25) | 287 (0.64) | 76 (0.43) | 713 (0.89) |
|  | Unspecified sepsis | 139 (0 29) | 222 (0.43) | 156 (0.35) | 41 (0.23) | 292 (0.37) |
|  | Malignant breast neoplasm | 93 (0.19) | 127 (0.24) | 121 (0.27) | 57 (0.32) | 152 (0.19) |
|  | Unspecified chest pain | 277 (0.58) | 400 (0.77) | 257 (0.57) | 64 (0.36) | 484 (0.61) |
|  | Syncope and collapse | 132 (0.28) | 171 (0.33) | 95 (0.21) | 36 (0.20) | 219 (0.27) |
| Incident COVID-19* | 0 | 44205 (92.4) | 48017 (92.6) | 41744 (92.8) | 16583 (92.8) | 73878 (92.7) |
|  | 1 | 3628 (7.58) | 3856 (7.43) | 3249 (7.22) | 1284 (7.19) | 5794 (7.27) |

Supplementary table 5 Median ITE for study medications, stratifed by the two pandemic waves. ACEIs: Angiotensin-converting-enzyme inhibitors, BBs: Beta blockers, CCBs: Calcium channel blockers, ITE: Individual treatment effect, THZs: Thiazide-like diuretic.

| Drug | label | levels | First Wave | Second Wave | p |
| --- | --- | --- | --- | --- | --- |
| ACEis | ITE | Median (IQR) | 0.0282 (-0.0156 to 0.0428) | 0.0364 (-0.0041 to 0.0452) | <0.001 |
| BBs | ITE | Median (IQR) | -0.0226 (-0.0738 to 0.0116) | -0.2035 (-0.2382 to -0.1817) | <0.001 |
| CCBs | ITE | Median (IQR) | -0.0367 (-0.0861 to -0.0254) | -0.2102 (-0.2759 to -0.2034) | <0.001 |
| Thiazides | ITE | Median (IQR) | 0.0083 (-0.0292 to 0.0189) | -0.0042 (-0.0117 to 0.0993) | <0.001 |
| Statins | ITE | Median (IQR) | 0.0238 (-0.0114 to 0.0315) | 0.0288 (0.0260 to 0.1386) | <0.001 |

Supplementary table 6 Weighted ATE for study medications, aggregated across both pandemic waves. ACEIs: Angiotensin-converting-enzyme inhibitors, BBs: Beta blockers, CCBs: Calcium channel blockers, THZs: Thiazide-like diuretic

| Drug | label | Overall Weighted ATE |
| --- | --- | --- |
| ACEis | Weighted ATE (SD) | 0.0097 (0.0552) |
| BBs | Weighted ATE (SD) | -0.0829 (0.0730) |
| CCBs | Weighted ATE (SD) | -0.0969 (0.0813) |
| Thiazides | Weighted ATE (SD) | 0.0427 (0.1083) |
| Statins | Weighted ATE (SD) | 0.0353 (0.0614) |

# **Supplementary Method**

## Main Components of the Transformer Model

The study employed a transformer-based neural network model, which was originally developed for natural language processing tasks (1) , to analyse the temporal patterns in health records . The core components of our model are detailed in Supplementary table 1.

**Preprocessing pipeline**

We create spatiotemporal sequences by flattening the drugs and hospital admissions time series into individual “sentences” with drug prescriptions and admission diagnoses being the “words”. In total, 186 unique ICD-10 codes and 305 unique BNF codes were provided to the model. Each word was subsequently tokenised and embedded as high-dimensional trainable vectors that allow the representation of concepts in numerical form, for which similar concepts have similar numerical representations. Instead of the positions in the sequence, the positional embedding index for each token was the month and year a drug or a diagnosis appeared in the 180-day study period.

**Transformer architecture**

When reading a sentence, each word is not processed in isolation, but rather consider its relationship to those before and after. Self-attention in a transformer neural network captures this essence, allowing them to "attend" to relevant parts of an input sequence when making predictions.

In the first step, temporal sequences were constructed by flattening time-series data into “sentences”, where drug prescriptions and admission diagnoses served as “words.” These words were tokenized and embedded as high-dimensional vectors. Temporal information was incorporated using positional embeddings that encoded the month and year of each event within the 180-day period. The addition of the word embedding vector and the positional embedding vector thus preserves both the semantic and chronological information of an item in the EHR. The resulted vectors were then fed into a multi-head attention layer of the transformer model to enable the identification of critical features and interactions (2) between different drug prescriptions or admissions at different time points across the study period, allowing for a more accurate analysis of treatment effects.

**Self Attention**

Self-attention computes the relevance of each word to every other word in a given sequence, using scaled dot products between query, key, and value vectors. The query and key vectors are derived from the word embeddings. The query vector represents the word that the model wants to focus on or retrieve information from the input. The key vector represents the word that the model wants to compare with the query vector. The dot product between a query and a key vector measures how similar they are. The dot products are then passed through a softmax function, which normalises the dot product values to obtain a probability distribution that sums up to one. The outputs from the softmax function are subsequently multiplied by the value vectors, which are usually the same as the key vector. The results are added together to produce the attention scores.

The self-attention output for each word is thus a new vector that combines the information from all the other words in the sentence, weighted by their relevance to the index word. The same process is repeated for the other words to generate the self-attention outputs for every word in the sentence. The result is a matrix of self-attention outputs, where each row corresponds to a word in the sentence, and each column corresponds to a dimension of the embedding. The matrix has the same shape as the original input matrix, but it has different values that reflect the attention mechanism. In this way, self-attention allows Transformers to capture dependencies between words, regardless of their distance from each other in the sentence. This is particularly useful for understanding the context and semantics of a sentence, making Transformers powerful tools for tasks like machine translation, text summarisation, and sentiment analysis.

In this study, the attention layer was configured to apply the input to itself (i.e., the same input). This self-attention operation allowed each element in the input sequence to attend to all other elements in the same sequence. By computing the attention scores between each pair of elements, our model learned to weigh and combine the relevant information from different drugs or admissions at different time within the study period when producing the output representations.

Finally, the transformer architecture was concatenated to a multi-layer perceptron (MLP), which is more efficient in modelling the static variables, to form the final model. The transformer model can be configured either to predict incident COVID-19 cases within 180 days from the onset of the first and second pandemic waves (classification task), or to estimate individual treatment effects (ITE) of a selected medication on the 180-day COVID-19 infection risk (regression task). This approach helps in capturing the complex dependencies between patient characteristics, medication use, and COVID-19 outcomes. An overview of our transformer-based neural network model methodology is visually represented in Supplementary figure 2.

### X-learner framework

The transformer model was then used as the base model for X-learner framework, which is a meta-algorithm specifically designed for studying causal inference and estimating individual treatment effects. (3) While most classification or regression models focus on finding patterns from the input features that are associated with the target variable, meta-learners aim to estimate the effect of an intervention on the outcome, also known as conditional average treatment effect (CATE). As such, while classification or regression models often struggle to distinguish between correlations and causal relationships in the data, meta-learners are designed to make causal inferences.

Traditionally, X-learner utilises base learners such as logistic regressions, random forests, or boosting algorithms to predict individual outcomes and treatment effects. However, these models often struggle to capture complex temporal and spatial interactions within the data.

In our study, we propose an innovative approach to incorporate the transformer model as the base learners into the X-learner framework.

The training of our Transformers-based-X learner algorithm included two stages. In the first stage, two transformer models were trained as classifiers to predict outcomes for the treated and control groups. The two transformer models were then applied to the opposite group to estimate counterfactual outcomes. ITEs were calculated as the difference between actual and counterfactual outcomes. In the second stage, two additional transformer models were built as regressors to predict ITEs for the treated and control groups, using the same input variables as the first-stage models. The two second-stage models were then applied to predict ITEs for the entire population. Final ITEs were calculated by averaging the two predictions from the second-stage models, weighted by propensity scores. The final average treatment effect (ATE) was calculated as the mean of ITEs across the population. A detailed description of the X-learner framework is described in Supplementary table 2. An overview of the X-learner framework is visualised in Supplementary figure 3.

## Transformer training process

The model consisted of 4 attention heads with 256 head size per head, 20% dropout, 32 hidden layer size for the feed forward network, and 32 embedding dimensions for each token.

Training the model involved a 0.001 learning rate, binary cross-entropy loss function, 30 epochs, and 32-sample batches. GPU processing was not used for the training of the model.

The convergence of the neural network was visualized through the training and validation loss over epochs (**Error! Reference source not found.**).

## Metrics and evaluation of model performance

The transformer model’s performance was evaluated using all hospital admissions recorded in SMR01 and medication dispenses from PIS during the 180-day period preceding each of the two COVID-19 pandemic waves, as well as static variables (age, sex, SIMD, and diabetes status). This approach was employed to assess the model's overall effectiveness in predicting incident COVID-19 in the next 180 days, taking into account all temporal dynamic interactions of medications and admissions, as well as static demographical information.

To ensure the robustness of the findings, a 5-fold cross-validation approach was employed. This method involves partitioning the data into 5 non-overlapping subsets and iteratively training the model on 4 subsets while validating it on the remaining subset. Performance metrics were calculated for each fold and averaged across the 5 iterations to provide a comprehensive assessment. The model was assessed using key performance metrics: Accuracy, F1 score, and AUPRC (Area Under the Precision-Recall Area Curve) across two distinct waves of data. (4-6). The F1 is the harmony of precision and recall, where recall measures the ratio of actual positive cases correctly identified, and precision reflects the ratio of predicted positive cases that are indeed correct. (7) PRAUC (Precision-Recall Area Under the Curve) was used in our study instead of ROCAUC, as it evaluates the proportion of true positives among the predicted positives and is thus considered more suitable for imbalanced classification tasks. (8)

Results were reported as mean ± standard deviation (SD). The model was evaluated using the default threshold of 0.5 to maintain equal importance between the two outcomes (COVID-19 positive or negative), and to prevent over- or under-estimation of the impact of the medications on either outcome.

We compared the performance of our transformer-based model to other traditional, machine learning, and deep learning classification methods including:

- Logistic regression
- XGBoost: A decision-tree based algorithm used for a wide range of medical applications.(4-6)
- Long short term memory: Another neural network architecture frequently used in natural language and timeseries classifications.(9, 10)

Performance for each submodel in the X-learners framework was similarly assessed using 5-fold cross validation. Stage 1 models were evaluated using accuracy, F1-score, and AUPRC. Stage 2 models estimate the continuous ITEs (3, 11, 12) and were thus evaluated based on mean absolute percentage error (MAPE) and mean absolute error (MAE).

# Supplementary Result

## Performance of X-learner subcomponents

In the stage 1 treated group, the model's accuracy ranged from 0.656 (±0.072) to 0.764 (±0.019) in the first wave and 0.686 (±0.020) to 0.748 (±0.021) in the second wave, while the control group's accuracy ranged from 0.707 (±0.025) to 0.767 (±0.050) in the first wave and 0.717 (±0.028) to 0.782 (±0.032) in the second wave. The treated group's F1 scores ranged from 0.664 (±0.021) to 0.756 (±0.023) in the first wave, and 0.667 (±0.029) to 0.753 (±0.024) in the second wave, compared to 0.704 (±0.031) to 0.787 (±0.045) and 0.694 (±0.022) to 0.801 (±0.045) in the control group. AUPRC values in the treated group ranged from 0.673 (±0.053) to 0.777 (±0.023) in the first wave, and 0.702 (±0.023) to 0.764 (±0.029) in the second wave, compared to 0.712 (±0.035) to 0.775 (±0.051) in the first wave, and 0.729 (±0.049) to 0.812 (±0.017) in the second wave for the control group (**Error! Reference source not found.** and **Error! Reference source not found.**).

For the stage 2 treated group, MAPE ranged from 0.091 (±0.004) to 0.199 (±0.012) in the first wave, and 0.056 (±0.004) to 0.213 (±0.006) in the second wave. For the stage 2 control group, MAPE ranged from 0.075 (±0.002) to 0.152 (±0.008) in the first wave, and 0.085 (±0.003) to 0.169 (±0.088) in the second wave (**Error! Reference source not found.** and **Error! Reference source not found.**).

# Supplementary references

1. Radford A, Narasimhan K, Salimans T, Sutskever I. Improving Language Understanding with Unsupervised Learning. Technical Report, OpenAI. 2018.

2. Vaswani A, Shazeer NM, Parmar N, Uszkoreit J, Jones L, Gomez AN, Kaiser L, Polosukhin I, editors. Attention is All you Need. Neural Information Processing Systems; 2017.

3. Künzel SR, Sekhon JS, Bickel PJ, Yu B. Metalearners for estimating heterogeneous treatment effects using machine learning. Proceedings of the National Academy of Sciences. 2019;116(10):4156-65.

4. Chen T, Guestrin C. XGBoost: A Scalable Tree Boosting System. Proceedings of the 22nd ACM SIGKDD International Conference on Knowledge Discovery and Data Mining2016. p. 785-94.

5. Yan L, Zhang H-T, Goncalves J, Xiao Y, Wang M, Guo Y, Sun C, Tang X, Jing L, Zhang M, Huang X, Xiao Y, Cao H, Chen Y, Ren T, Wang F, Xiao Y, Huang S, Tan X, Huang N, Jiao B, Cheng C, Zhang Y, Luo A, Mombaerts L, Jin J, Cao Z, Li S, Xu H, Yuan Y. An interpretable mortality prediction model for COVID-19 patients. Nature Machine Intelligence. 2020;2(5):283-8.

6. Tran TQB, Lip S, du Toit C, Kalaria TK, Bhaskar RK, O’Neil AQ, Graff B, Hoffmann M, Szyndler A, Polonis K, Wolf J, Reddy S, Narkiewicz K, Dasgupta I, Dominiczak AF, Visweswaran S, McCallum L, Padmanabhan S. Assessing Machine Learning for Diagnostic Classification of Hypertension Types Identified by Ambulatory Blood Pressure Monitoring. CJC Open. 2024;6(6):798-804.

7. Powers DMW. Evaluation: from precision, recall and F-measure to ROC, informedness, markedness and correlation. ArXiv. 2011;abs/2010.16061.

8. Brock G, Saito T, Rehmsmeier M. The Precision-Recall Plot Is More Informative than the ROC Plot When Evaluating Binary Classifiers on Imbalanced Datasets. Plos One. 2015;10(3).

9. Chauhan S, Vig L. Anomaly detection in ECG time signals via deep long short-term memory networks. 2015 IEEE International Conference on Data Science and Advanced Analytics (DSAA)2015. p. 1-7.

10. Thorsen-Meyer H-C, Nielsen AB, Nielsen AP, Kaas-Hansen BS, Toft P, Schierbeck J, Strøm T, Chmura PJ, Heimann M, Dybdahl L, Spangsege L, Hulsen P, Belling K, Brunak S, Perner A. Dynamic and explainable machine learning prediction of mortality in patients in the intensive care unit: a retrospective study of high-frequency data in electronic patient records. The Lancet Digital Health. 2020;2(4):e179-e91.

11. Curth A, Schaar Mvd. Nonparametric Estimation of Heterogeneous Treatment Effects: From Theory to Learning Algorithms. In: Arindam B, Kenji F, editors. Proceedings of The 24th International Conference on Artificial Intelligence and Statistics; Proceedings of Machine Learning Research: PMLR; 2021. p. 1810--8.

12. Ling Y, Upadhyaya P, Chen L, Jiang X, Kim Y. Emulate randomized clinical trials using heterogeneous treatment effect estimation for personalized treatments: Methodology review and benchmark. Journal of Biomedical Informatics. 2023;137.
